# Jorge Cervós-Navarro 1930–2021

**DOI:** 10.1007/s00401-022-02456-x

**Published:** 2022-07-23

**Authors:** Gisela Stoltenburg-Didinger, Hans-Hilmar Goebel

**Affiliations:** Charite–Universitätsmedizin Berlin, Charitéplatz 1, 10117 Berlin, Germany

Jorge (in Spanish) or Jordi (in Catalan) Cervós-Navarro, the three-decade-long chairman and founder of the Institute of Neuropathology at the Free University of Berlin, was born in Barcelona/Spain on the 9th of January 1930 and died there on the 14th of November, 2021.


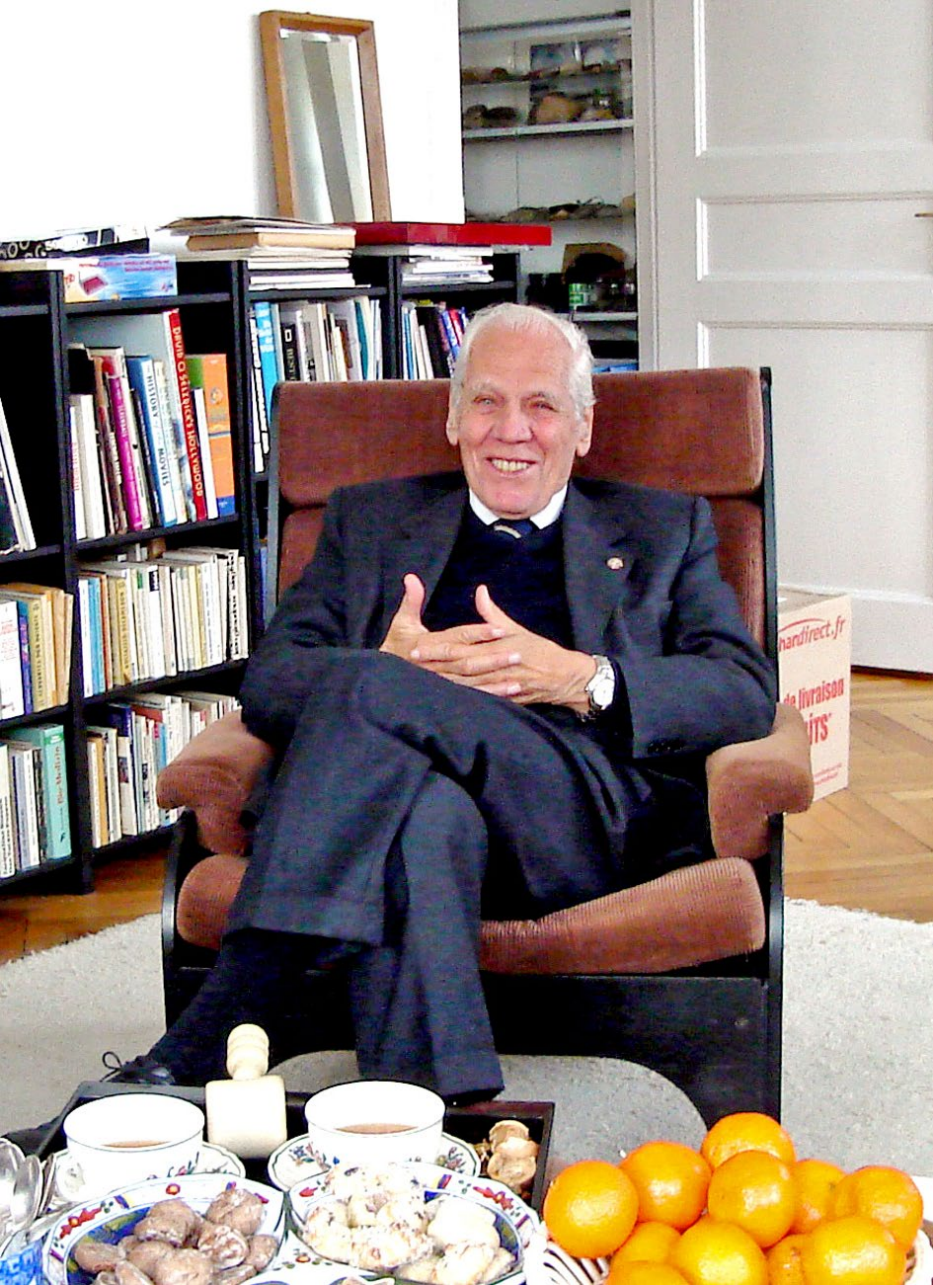
 Jorge Cervós-Navarro

As a precaution during the Spanish Civil War, 1936–1939, his parents sent him to maternal relatives in the Pyrenees which generated a life-long love for mountains, even, as he loved to recount, as a shepherd of goats and sheep, an episode he most fondly remembered when stricken late in life by immobilizing Parkinson disease. He returned to Barcelona for further schooling and the subsequent study of medicine which he began at the age of 16 years. After his medical graduation, he added three more years of religious studies at the University of Zaragossa.

He had an optimistic outlook on life: “I have been born on the sunny side of life, and when a shadow approaches I just move one step aside”.

He first went into psychiatric training at Innsbruck/Austria, but only four months later he started specialist training in neuropathology in Bonn/Germany. Remarkably successful in academic life, he was offered, in 1968, at the age of only 38 years, the newly created chair in Neuropathology at the Free University of Berlin.

Over the following years, he established the largest Institute of Neuropathology in the country and, perhaps, even in Europe. Unlike others among his faculty colleagues who sometimes used a first full professorship in West Berlin as a stepping stone to more traditional university chairs in West Germany, he remained in Berlin as head of his institute for the subsequent 30 years until mandatory retirement.

He trained many young doctors from Germany and from Spain, fertilizing both German and Spanish neuropathology and neurology, and he was host to several highly reputed neuropathologists from abroad who spent sabbaticals at his institution, especially from the USA through a Humboldt Fellowship, e.g., Edward P. Richardson, Julio Garcia, and James Nelson.

However, he never forgot where he came from. He remained a Spanish citizen and never became a German one although he had achieved the highest professional rank in the German academic hierarchy. He brought knowledge of his institute, his field, his University and his city of Berlin abroad, especially to institutions in countries behind the Iron Curtain. He predicted — based on his conversational experiences and his personal as well as professional observations long before the collapse of communism—its decline and final disintegration. He was bestowed with several honorary doctorates, i.e., from Barcelona, Zaragossa, Madrid, his home country, Hannover/Germany, Sagansk/Russia, Thessaloniki/Greece, and Tokushima/Japan.

Jorge Cervós-Navarro was a gifted organizer and academic entrepreneur, for instance he organized one of the early European Congresses of Neuropathology in Berlin which in those times were not arranged by scientific or lay societies, but single-handedly by individuals, such as Kurt Jellinger of Vienna, Jorge Cervós-Navarro of Berlin and Miroslaw J. Mossakowski of Warsaw without any back-up or support, before the European Confederation of Neuropathological Societies (EuroCNS) took the European Congress under its wings. He loved to organize and edit books and scientific proceedings, some fifteen altogether [1–15]. He was an early ardent and later accomplished electron microscopist inspired by the work presented by his American colleagues. He liked to travel, professionally and para-professionally, and his travels brought him around the globe, especially in narrow reach, across the Iron Curtain where he promoted Neuropathology.

Finally, obeying the mandatory rule of German academic retirement, he withdrew to his hometown, Barcelona, and became the first Rector (president/ vice-chancellor) of the newly established International University of Catalunya and thereafter its global ambassador. Toward the end of his life, he wrote an autobiography, on his professional career in Catalan [16]. This and his professional books and papers as well as his Institute of Neuropathology in Berlin are a legacy and testimony of a man and neuropathologist who found his firm place in the history of Neuropathology.
